# Development of a nomogram for the prediction of complicated appendicitis during pregnancy

**DOI:** 10.1186/s12893-023-02064-w

**Published:** 2023-07-01

**Authors:** Xiaosong Zheng, Xiaojun He

**Affiliations:** Department of General Surgery, Maternal and Child Health Hospital of Hubei Province, Tongji Medical College, Huazhong University of Science and Technology, NO.745 Wuluo Road, Hongshan District, Wuhan City, Hubei Province 430070 P.R. China

**Keywords:** Nomogram, Complicated appendicitis, Pregnancy

## Abstract

**Background:**

Complicated appendicitis during pregnancy directly affects the clinical prognosis of both mother and fetus. However, accurate identification of complicated appendicitis in pregnancy is fraught with various challenges. The purpose of this study was to identify the risk factors and to develop a useful nomogram to predict complicated appendicitis during pregnancy.

**Methods:**

This retrospective study involved pregnant women who underwent appendectomy at the Maternal and Child Health Hospital of Hubei Provincial from May 2016 to May 2022 and who ultimately had histopathological confirmed acute appendicitis. Univariate and multivariate logistic regression were applied to analyze clinical parameters and imaging features as a way to identify risk factors. Then, nomogram and scoring systems predicting complicated appendicitis in pregnancy were constructed and evaluated. Finally, the potential non-linear association between risk factors and complicated appendicitis was analyzed using restricted cubic splines.

**Results:**

Three indicators were finally identified for the construction of the nomogram: gestational weeks, C-reactive protein (CRP), and neutrophil percentage (NEUT%). To improve the clinical utility, the gestational weeks were divided into three periods (first trimesters, second trimesters, and third trimesters), while the optimal cut-offs for CRP level and NEUT% were found to be 34.82 mg/L and 85.35%, respectively. Multivariate regression analysis showed that third trimesters (P = 0.013, OR = 16.81), CRP level ≥ 34.82 mg/L (P = 0.007, OR = 6.24) and NEUT% ≥85.35% (P = 0.011, OR = 18.05) were independent risk factors for complicated appendicitis. The area under the ROC curve (AUC) of the nomogram predicting complicated appendicitis in pregnancy was 0.872 (95% CI: 0.803–0.942). In addition, the model was shown to have excellent predictive performance by plotting calibration plots, Decision Curve Analysis (DCA), and clinical impact curves. When the optimal cut-off point of the scoring system was set at 12, the corresponding AUC, sensitivity, specificity, Positive Likelihood Ratio (PLR), Negative Likelihood Ratio (NLR), Positive Predictive Value (PPV), and Negative Predictive Value (NPV) values were AUC: 0.869(95% CI: 0.799–0.939),100%, 58.60%, 2.41, 0, 42%, and 100%, respectively. The restricted cubic splines revealed a linear relationship between these predictors and complicated appendicitis during pregnancy.

**Conclusions:**

The nomogram utilizes a minimum number of variables to develop an optimal predictive model. Using this model, the risk of developing complicated appendicitis in individual patients can be determined so that reasonable treatment choices can be made.

**Supplementary Information:**

The online version contains supplementary material available at 10.1186/s12893-023-02064-w.

## Background

Acute appendicitis (AA) during pregnancy is a common surgical emergency abdomen with an incidence of approximately 1 in 1700 [1–3]. Based on clinical features, pathological findings, and disease prognosis, AA is classified into complicated appendicitis (appendiceal abscess, gangrenous, and perforated appendicitis) and uncomplicated appendicitis (simple appendicitis, and suppurative appendicitis) [4]. Previous studies have consistently advocated surgical treatment of AA during pregnancy [5, 6]. However, a growing number of studies have found that non-surgical treatment is an acceptable option for patients with uncomplicated appendicitis during pregnancy [7–13]. In patients with complicated appendicitis, delayed diagnosis and treatment significantly increases the risk of maternal complications and fetal loss [14–18]. Therefore, it is particularly important to distinguish between complicated and uncomplicated appendicitis, both in the choice of treatment options and in the assessment of prognosis. For non-pregnant patients, researchers have developed scoring models to identify complicated appendicitis [19]. However, no predictive models have been developed for patients with complicated appendicitis during pregnancy. The purpose of this study was to investigate risk factors and to develop a useful nomogram and scoring system for individualized prediction of complicated appendicitis during pregnancy.

## Methods

### Study design and study subjects

We conducted a retrospective study in a large tertiary teaching hospital in Wuhan, Hubei Province, China, which complied with the revised Declaration of Helsinki and was approved by the Medical Ethics Committee of Maternal and Child Health Hospital of Hubei Province. Because the study was based on a retrospective non-interventional design, the requirement to obtain informed consent was waived (Medical Ethics Committee of Maternal and Child Health Hospital of Hubei Province). All analyses were performed based on the Transparent Reporting of a Multivariable Prediction Model for Individual Prognosis or Diagnosis statement [20].

To construct a nomogram predicting complicated appendicitis in pregnancy, we collected pregnant women who underwent appendectomy from May 2016 to May 2022 at the Maternal and Child Health Hospital of Hubei Provincial and who ultimately had acute appendicitis confirmed by histopathology. Inclusion criteria were pregnant women who met the diagnosis of AA and were between 18 and 40 years of age. The exclusion criteria were as follows. (1) appendiceal abscess; (2) combined acute inflammation at other sites; (3) combined immune system disorders; (4) combined diabetes; (5) anti-infective treatment before admission; (6) missing data.

### Pathological assessment and classification

Based on the operative report and the histopathological findings of the appendix, AA was classified as complicated or uncomplicated. Complicated appendicitis was defined as gangrenous or perforated appendicitis, whereas simple and suppurative appendicitis was considered uncomplicated (Table 1) [4, 19]. When there is a conflict between the surgical findings and the pathology report, the surgical findings play a decisive role.

**Table 1:** Diagnostic criteria of CA (Complicated Appendicitis) and UA (Uncomplicated Appendicitis)

	Macroscopic appearances	Microscopic appearances
**Normal appendix**		
Normal underlying pathology	No visible changes	Absence of any abnormality
Acute intraluminal inflammation	No visible changes	Luminal neutrophils only with no mucosal abnormality
Acute mucosal/submucosal inflammation	No visible changes	Mucosal or submucosal neutrophils and/or ulceration
**Simple, non-perforated appendicitis**		
Suppurative/phlegmonous	Congestion, colour changes, increased diameter, exudate, pus	Transmural inflammation, ulceration, or thrombosis, with or without extramural pus
**Complex appendicitis**		
Gangrenous	Friable appendix with purple, green, or black colour changes	Transmural inflammation with necrosis
Perforated	Visible perforation	Perforation; not always visible in microscope
Abscess (pelvic/abdominal)	Mass found during examination or abscess seen on preoperative imaging; or abscess found at surgery	Transmural inflammation with pus with or without perforation

### Variables associated with complicated appendicitis

Due to the paucity of studies on complicated appendicitis in pregnancy, we chose to identify predictors suspected to be associated with complicated appendicitis in pregnancy by searching the general population medical literature, which contains clinical manifestations and ancillary tests. We also took into account the general situation of pregnant women and identified and selected the following clinical features for analysis: age, gestational age, pulse, history of AA, parturition, duration of abdominal pain, temperature (TEMP, ℃), vomiting, anorexia, diarrhea, shifting pain in the right lower quadrant, rebound pain in the right lower quadrant, white blood cell (WBC) count, neutrophil-to-lymphocyte ratio (NLR), neutrophil percentage (NEUT%), C-reactive protein (CRP) level, total bilirubin (TBIL), and platelet (PLT) count. the gestational weeks were divided into three periods (first trimesters: ≤13^+ 6^ weeks, second trimesters: 14^+ 0^-27^+ 6^ weeks, and third trimesters: 28^+ 0^-40^+ 6^ weeks). The ultrasound features which are associated with complicated appendicitis are periappendiceal fluid and appendicolith. All of the above variables were retrospectively collected at the time of patient admission through structured case notes.

### Statistical analysis

All data were analyzed using R software for Windows (version 4.2.1). Normally distributed data for continuous variables are expressed using standard deviation (SD) ± mean, and non-normally distributed data are described by median and interquartile range (IQR). categorical variables are expressed as the number of cases (percentage, %). The variables of interest were assessed by univariate logistic regression analysis to identify independent risk factors for complicated appendicitis during pregnancy. All variables associated with complicated appendicitis in pregnancy are candidates for stepwise multivariate analysis. To improve the clinical applicability of the nomogram, optimal cut-off values for continuous variables were determined based on receiver operating characteristic (ROC) curves and the Youden index.

Nomogram was developed using the R version of the rms package according to the results of multivariate logistic regression analysis. The nomogram converts each regression coefficient into a score from 0 to 10. The variable with the highest β coefficient (absolute value) is assigned a score of 10. The points corresponding to all independent variables are added to obtain the total number of points, which are converted to predicted probabilities. The area under the ROC curve (AUC) and calibration (rms package, Hosmer–Lemeshow test (HLtest.R)) with 1000 bootstrap samples to decrease the overfit bias were used to estimate the predictive performance of the nomogram [21, 22]. Clinical Impact Curves (CIC) curves and Decision Curve Analysis (DCA) curves were used to assess the clinical utility of the nomograms, which were plotted by using the rmda software package [23]. Finally, the nomogram was translated into a clinically applicable scoring system called the nomogram risk score. The score corresponding to each variable in the scoring system is an integer and these scores are obtained by rounding off the corresponding values in the nomogram. The threshold values of the scoring system were then determined, while the discrimination and calibration of the scoring system were evaluated.

Finally, we estimated a possible non-linear relationship between risk factors for continuous variables and complicated appendicitis by restricted cubic splines [24]. It was analyzed and plotted by using the rms package. The 5th, 35th, 65th, and 95th percentiles were set as the knots.

## Results

### Participant characteristics of acute appendicitis during pregnancy

**Fig. 1:**
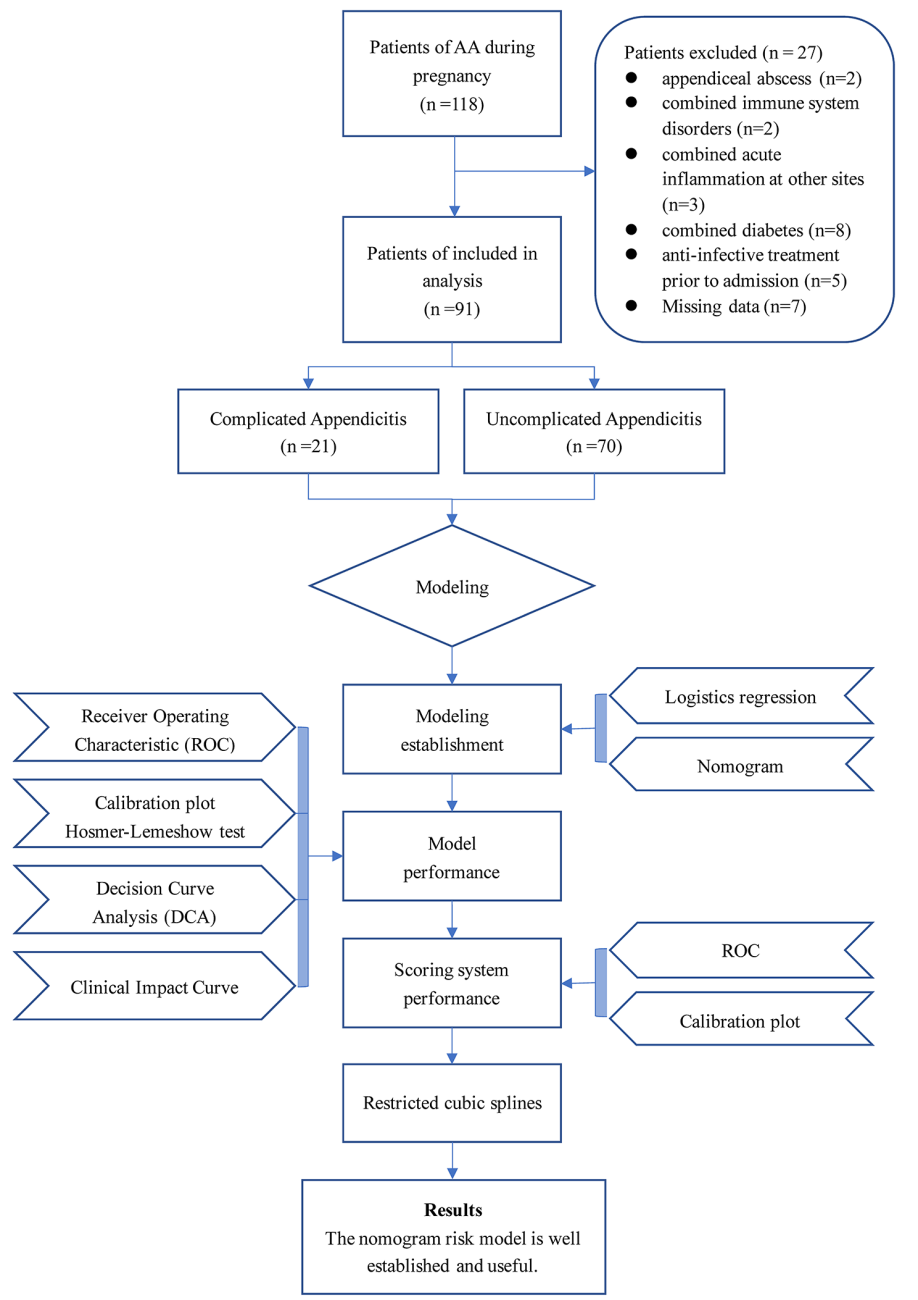
Study flow diagram

A total of 118 patients who underwent appendectomy during pregnancy were included for observation, of which 27 patients were excluded based on the exclusion criteria (Fig. 1). Of these, 91 patients who met the inclusion criteria were enrolled, and 21 and 70 patients were divided into complicated appendicitis and uncomplicated appendicitis cohorts, respectively. The baseline characteristics of acute appendicitis during pregnancy are summarized in eTable.1 in the Supplement.

### Identification of independent risk factors

Univariate regression analysis found four variables to be statistically significant. However, in this multivariate regression analysis, data from the uncomplicated and complicated appendicitis groups were compared using stepwise backward logistic regression analysis, and the results suggested that gestational weeks, CRP, and NEUT% were statistically significant independent risk factors (eTable.2 in the Supplement). To improve the clinical utility, the gestational weeks were divided into three periods (first trimesters, second trimesters, and third trimesters), while the optimal cut-offs for CRP level and NEUT% were found to be 34.82 mg/L and 85.35%, by ROC curves, respectively.

Further multivariate regression analysis showed that third trimesters (P = 0.013, OR = 16.81), CRP level ≥ 34.82 mg/L (P = 0.007, OR = 6.24) and NEUT% ≥85.35% (P = 0.011, OR = 18.05) were independent risk factors for complicated appendicitis (Table 2).

**Table 2:** Multivariate Logistic Regression Analysis of the Association between Variables and Complicated Appendicitis during Pregnancy

Variables	Multivariate analysis	
	OR (95% CI)	*P* value
Trimesters		
First	1.0 (Reference)	
Second	4.64(0.85–38.99)	0.104
Third	16.81 (2.15-201.76)	0.013
NEUT%		
<85.35%	1.0 (Reference)	
≥ 85.35%	18.05 (2.86-364.82)	0.011
CRP(mg/L)		
<34.82	1.0 (Reference)	
≥ 34.82	6.24(1.75–25.84)	0.007

NEUT%, neutrophil percentage; CRP, C-reactive protein

### Nomogram and model performance

**Fig. 2:**
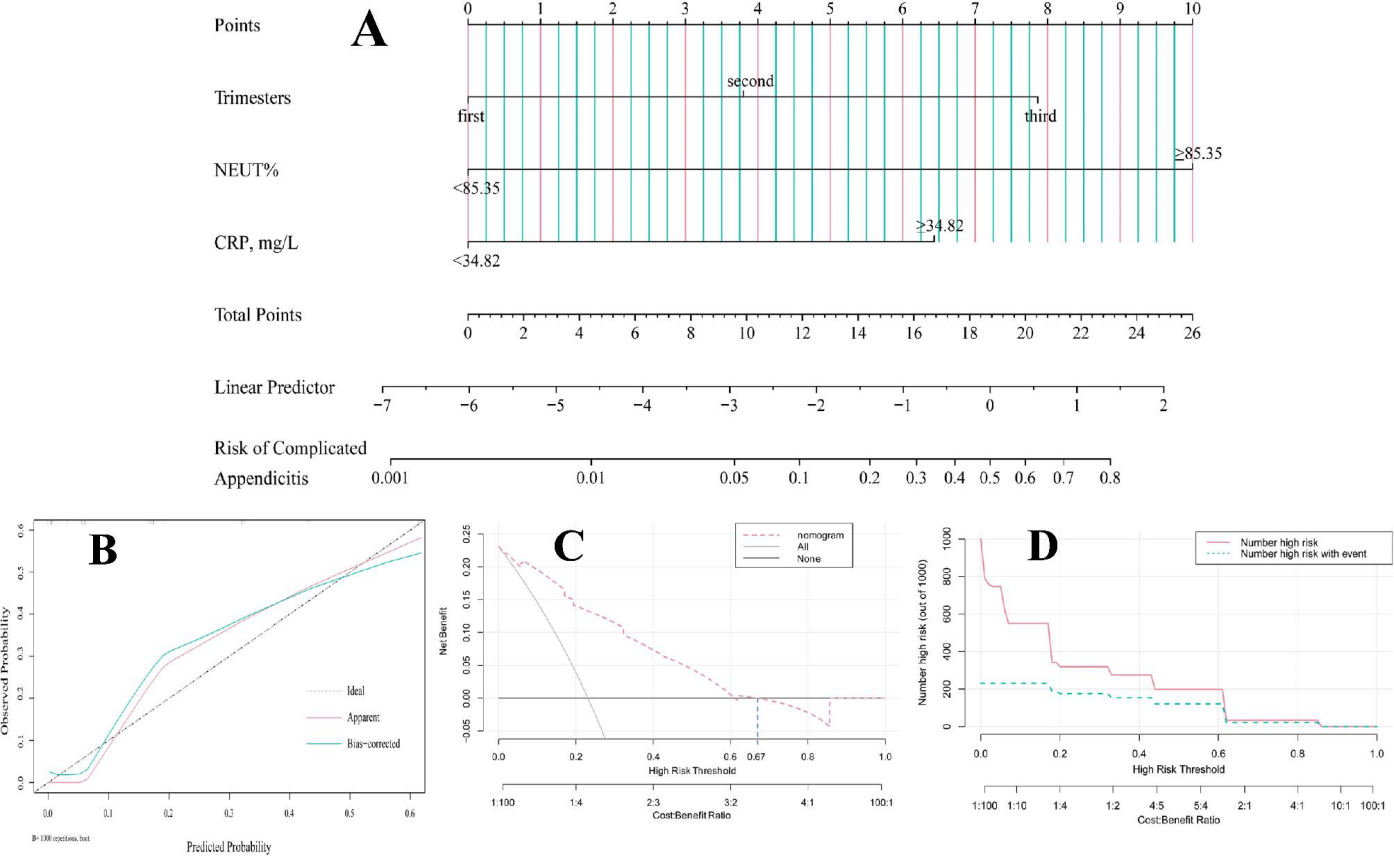
**A**, Nomogram to predict complicated appendicitis during pregnancy (Trimesters, NEUT%, and CRP). **B**, Calibration curve: The x-axis reflexes the predicted risk. The y-axis reflexes the actual risk of complicated appendicitis. The diagonal dotted represents a perfect prediction by an ideal model. The red and blue lines represent the performance of the nomogram, of which a closer fit to the diagonal dotted line represents a better prediction. **C**, Decision Curve Analysis (DCA): The vertical axis represents the value of net benefit, and the horizontal axis represents the threshold level. If the threshold probability is 0–64%, then the use of this nomogram is beneficial in clinical practice. **D**, Clinical Impact Curves (CIC): the red curve (Number high risk) indicates the number of individuals classified as positive (high-risk) by the model at each threshold probability; the blue curve (Number high risk with event) is the number of true positives at each threshold probability. the CIC visually indicates that the nomogram has a high net clinical benefit.

Backward stepwise regression analysis using AIC in a logistic regression analysis model eventually revealed a significant association between the following 3 variables and complicated appendicitis: third trimesters, CRP ≥ 34.82 mg/L, and NEUT% ≥85.35%. Nomogram to predict complicated appendicitis during pregnancy are shown in Fig. 2A.

The nomogram clearly displays that each predictor corresponds to a different score. The total point was the sum of the points of three predictors for each patient. The bottom of the nomogram displays the relationship between the total score and the probability of complicated appendicitis. The higher the total score based on the sum of the assigned points for each factor in the nomogram, the more likely complicated appendicitis was indicated.

The area under the ROC curve was used to assess the discriminatory ability of the nomogram, and the results showed that the AUC of the nomogram was 0.872 (95% CI: 0.803–0.942), which indicates moderately high performance (eFigure.2 in the Supplement). A calibration plot and Hosmer–Lemeshow test were adopted to calibrate the predictive model. The analysis showed good consistency between predicted and actual probabilities (p = 0.869) (Fig. 2B). In addition, DCA was used to evaluate the clinical utility of the nomogram. As depicted in Fig. 2C, if the threshold probability is 0–64%, patients with complicated appendicitis during pregnancy would benefit more from the nomogram than with treat all or no treatment conditions. Based on the results of DCA, CIC was further developed to assess the clinical utility of the nomograms. CIC revealed that the predicted probabilities matched well with the actual probabilities (Fig. 2D).

### Diagnostic performance of the scoring system

**Fig. 3:**
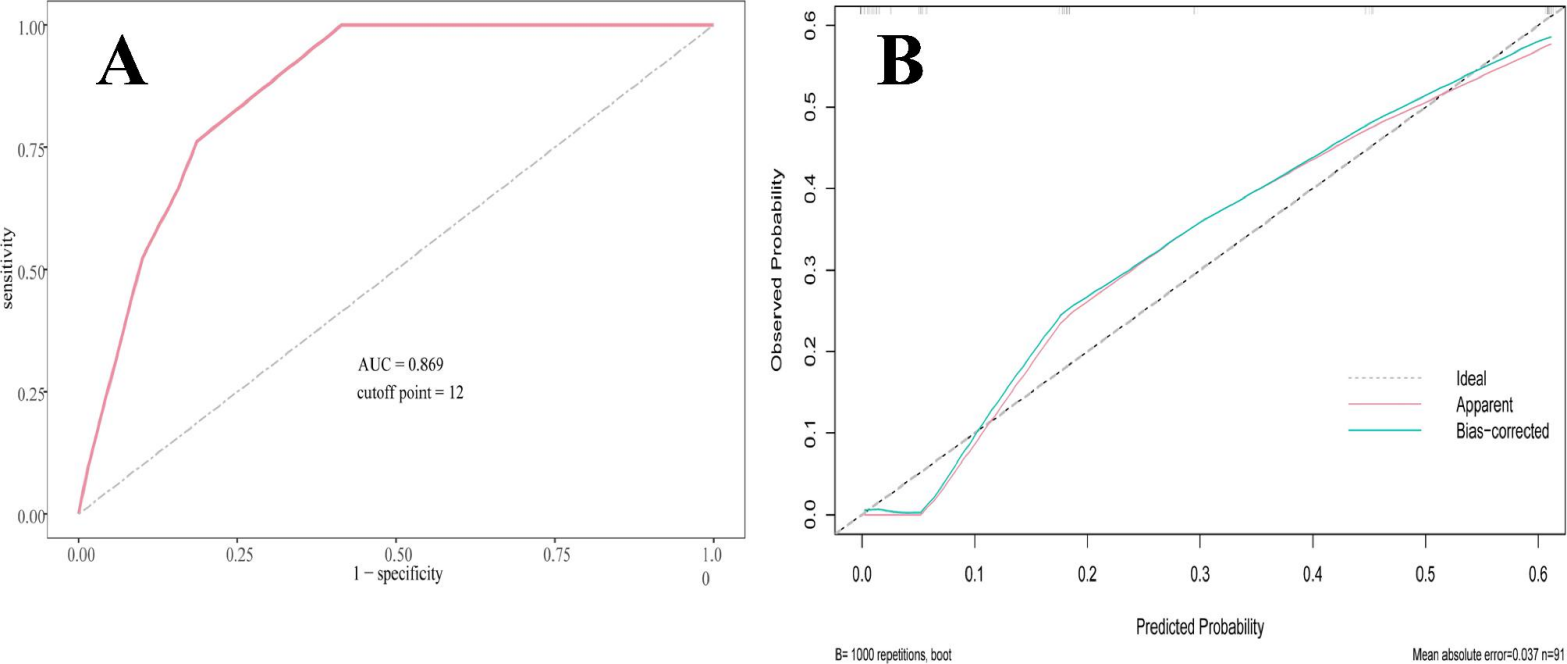
**A**, ROC curves validate the discriminatory power of the scoring system predictive for complicated appendicitis. AUC: 0.869. **B**, Calibration curve of the scoring system for predicting complicated appendicitis during pregnancy

We modified the nomogram to a scoring system with integer points to facilitate better use in clinical practice: First trimesters (0 points), Second trimesters (4 points), Third trimesters (8 points), NEUT% <85.35% (0 points), NEUT% ≥85.35% (10 points), CRP<34.82 mg/L (0 points), CRP ≥ 34.82 mg/L (6 points) (Table 3). Based on the scores of the above variables, the total score for each patient was calculated and then the ROC curve was plotted (Fig. 3A). The results show that the scoring system has shown excellent ability in differentiating complicated and uncomplicated appendicitis (AUC: 0.869, 95% CI: 0.799–0.939). When the cut-off is set at 12, the likelihood that the patient will be diagnosed with complicated appendicitis is greatly increased if the total score exceeds 12. However, when the total score is below 12, patients are less likely to be diagnosed with complicated appendicitis. When the optimal cut-off point was set at 12, the corresponding sensitivity, specificity, PLR, NLR, PPV, and NPV values were 100%, 58.60%, 2.41, 0, 42%, and 100%, respectively (Table 4). At the same time, the scoring system also shows good calibration, which means that the predicted probabilities of the scoring system are highly consistent with the actual probabilities (p = 0.909) (Fig. 3B).

**Table 3:** Scoring system for Complicated Appendicitis during Pregnancy

Variables	scores
Trimesters	
First	0
Second	4
Third	8
NEUT%	
<85.35%	0
≥ 85.35%	10
CRP (mg/L)	
<34.82	0
≥ 34.82	6

**Table 4:** ROC analysis of the scoring system for identifying Complicated Appendicitis during Pregnancy

Cut-off score	Youden index	Sensitivity	95% CI	Specificity	95% CI	PLR
> 7	0.31	1.00	0.85–1.00	0.31	0.22–0.43	1.46
> 9	0.33	1.00	0.85–1.00	0.33	0.23–0.45	1.49
> 12	0.59	1.00	0.85–1.00	0.59	0.47–0.69	2.41
> 15	0.57	0.76	0.55–0.89	0.81	0.71–0.89	4.10
> 17	0.51	0.67	0.45–0.83	0.84	0.74–0.91	4.24

CI = confidence interval; PLR = positive likelihood ratio

### Restricted cubic splines of complicated appendicitis during pregnancy

**Fig. 4:**
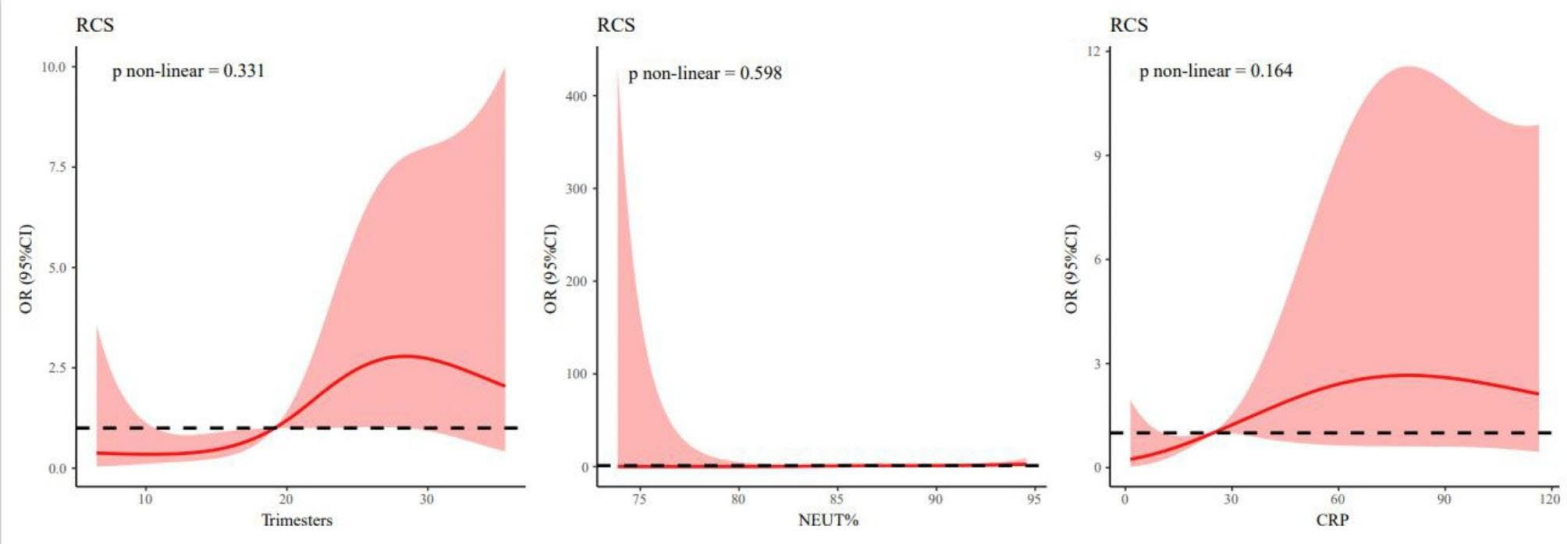
Restricted cubic splines of complicated appendicitis during pregnancy: Trimesters, NEUT% and CRP.

To further clarify the relationship between predictors and complicated appendicitis, we performed a restricted cubic splines analysis. As shown in Fig. 4, the relationship between all these predictors and complicated appendicitis in pregnancy was linear (P for non-linear > 0.05), which further proves that these predictors of continuous variables have excellent predictive performance.

## Discussion

Globally, the annual incidence of AA is 96.5 to 100 cases per 100,000 adult population [25, 26]. In the female population, the lifetime prevalence is 6.7% [27]. AA often occurs in the second trimester [2]. For the purpose of clinical management, AA is often classified as uncomplicated and complicated appendicitis. The rate of fetal abortion in patients with simple appendicitis is approximately 1.5% and increases significantly in the presence of combined complications (e.g. appendiceal perforation, gangrene, abscess, peritonitis, etc.) [28]. Recently, 2 studies have found that conservative treatment is an ideal approach for patients with uncomplicated appendicitis during pregnancy [9, 10]. However, immediate surgery may be the treatment strategy of choice for patients with complicated appendicitis [14]. Therefore, the diagnosis of complicated appendicitis is a very important task, both in terms of the choice of treatment options and in determining the prognosis of the disease. However, the diagnosis remains ambiguous in many patients, which is one of the most challenging issues.

To our knowledge, this is the first nomogram graph that can be used to accurately predict complicated appendicitis during pregnancy. It includes 3 variables: week of gestation and two routinely obtained laboratory tests. For convenience, we transformed the nomogram into a simple scoring system. The AUC of this scoring system was 0.869. When the optimal cut-off point was set at 12, the corresponding sensitivity and specificity were 100% and 58.60%, respectively. Therefore, it is a quick and low-cost tool to aid in the diagnosis of AA during pregnancy.

Among the currently available prediction tools, the nomogram is an accessible tool with high accuracy and good discrimination in predicting results [29]. By screening for risk factors, we eventually developed a nomogram for identifying complicated appendicitis in pregnancy, and it has good accuracy, discrimination, and clinical decision curve. The nomogram included Trimesters, NEUT%, and CRP.

The specificity of the pregnancy period makes the diagnosis of AA during pregnancy relatively difficult [30]. The typical presentation of AA is metastatic right lower abdominal pain with or without gastrointestinal symptoms. In this study, right lower abdominal pain was the most common clinical manifestation of AA in pregnancy. 54.9% of patients with AA in pregnancy presented with typical metastatic right lower abdominal pain. This is similar to the percentage reported in previous literature [31]. In this study, metastatic right lower abdominal pain was present in 57.1% of the complicated appendicitis group, which was not different from the uncomplicated group. In addition, digestive symptoms (e.g., nausea, vomiting, diarrhea, etc.) and right lower abdominal rebound pain did not differ between the two groups. This finding suggests that the clinical presentation does not seem to be of particular value in differentiating complicated appendicitis in pregnancy. This may be related to the specific physiological and anatomical alterations during pregnancy (e.g., pregnancy reaction, uterine enlargement, and abdominal wall laxity) [31]. On the effect of gestational week on complicated appendicitis. The investigators observed a significant increase in the incidence of complicated appendicitis with increasing weeks of gestation [32]. This was also confirmed in the present study. Therefore, the gestational week was included in the nomogram and scoring system for predicting complicated appendicitis during pregnancy.

Although physiological leukocytosis and neutrophilia are often present during pregnancy, they still have practical value in the diagnosis of AA in pregnancy [33–35]. In this study, leukocyte and neutrophil ratios were detected to be elevated significantly in patients with complicated appendicitis during pregnancy. However, in the final model, the neutrophil ratio alone was discovered to be an independent risk factor for complicated appendicitis. When the neutrophil ratio was ≥ 85.35%, the risk of complicated appendicitis was increased 18.05 times. CRP is a valuable inflammatory predictor of AA [36]. When CRP was ≥ 34.82 mg/L, we observed 6.24 times increase in the risk of complicated appendicitis.

Abdominal ultrasound and MRI play an important role in the diagnosis of AA during pregnancy. Ultrasound is the preferred screening method when AA is suspected during pregnancy [37, 38]. However, several studies have demonstrated a high failure rate of ultrasonography for the diagnosis of AA in pregnancy [39–41]. Therefore, the American College of Radiology recommends MRI as the method of choice for negative ultrasound examinations [38]. Scoring models incorporating imaging features have been developed to identify patients with complicated appendicitis in the general population [19, 42]. In this study, a retrospective investigation of ultrasound parameters (periappendiceal fluid and appendicolith) in patients with appendicitis during pregnancy revealed no advantage of abdominal ultrasonography in differentiating complicated appendicitis. Two of these patients were suspected of having AA in pregnancy, and we performed an abdominal MRI when the abdominal ultrasound was negative, and they were finally diagnosed with AA. This implies that ultrasound has no significant advantage in the diagnosis of appendicitis in pregnancy or the identification of complicated appendicitis. However, the role of abdominal MRI in identifying complicated appendicitis in pregnancy needs to be further investigated.

The specificity of appendicitis in pregnancy led to fewer patients meeting the study’s inclusion criteria, resulting in a relatively small sample size. Therefore, only internal validation of the model was performed, while external validation was not possible. We hope that more research scholars will validate our model in the future. In addition, although we have analyzed many potential risk factors, we still cannot completely exclude that some unadjusted confounding may have an impact on the results. Also, it cannot be excluded that some unknown variables may help to improve the accuracy of the model.

## Electronic supplementary material

Below is the link to the electronic supplementary material.

Additional File 1: The cut-offs of the CRP level and the NEUT%

Additional File 2: The nomogram of ROC

Additional File 3: The baseline data of CA (Complicated Appendicitis) group and UA (Uncomplicated Appendicitis) group

Additional File 4: Logistic regression model of the association between variables and complicated

## Data Availability

The datasets of the current study are available from the corresponding author upon reasonable request.
