# Supplementary figures and images for: Development of a nomogram for the prediction of complicated appendicitis during pregnancy
Source: BMC Surg. 2023 Jul 1;23:188. doi: 10.1186/s12893-023-02064-w (PMC10315032; doi:10.1186/s12893-023-02064-w)

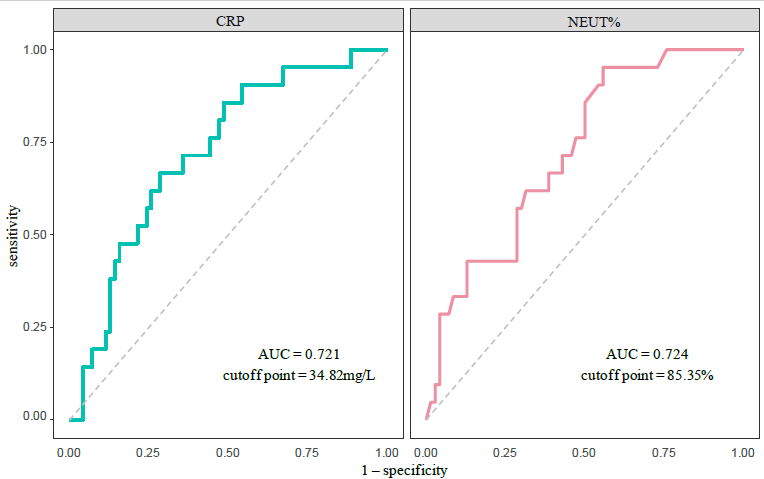


eFigure1. The cut-offs of the CRP level and the NEUT%.

Supplement: Supplementary file 1 — Additional File 1: The cut-offs of the CRP level and the NEUT% [file 12893_2023_2064_MOESM1_ESM.docx]

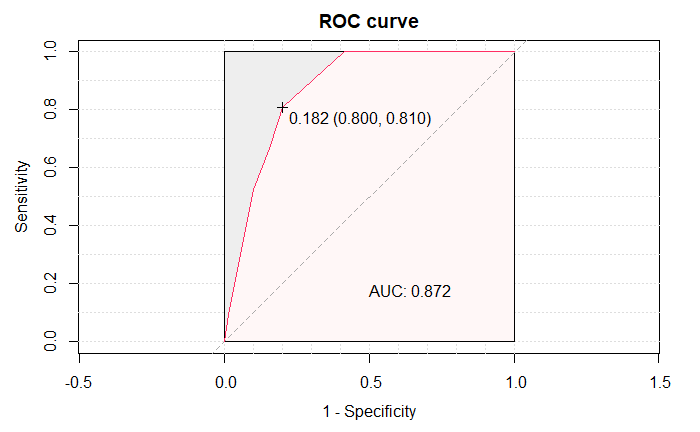


eFigure.2. The nomogram of ROC.

Supplement: Supplementary file 2 — Additional File 2: The nomogram of ROC [file 12893_2023_2064_MOESM2_ESM.docx]
