# Supplementary material for: Development of a nomogram for the prediction of complicated appendicitis during pregnancy
Source: BMC Surg. 2023 Jul 1;23:188. doi: 10.1186/s12893-023-02064-w (PMC10315032; doi:10.1186/s12893-023-02064-w)
Supplement: Supplementary file 3 — Additional File 3: The baseline data of CA (Complicated Appendicitis) group and UA (Uncomplicated Appendicitis) group [file 12893_2023_2064_MOESM3_ESM.docx]

eTable.1 The baseline data of CA (Complicated Appendicitis) group and UA (Uncomplicated Appendicitis) group

|  | **Total** | **CA group** | **UA group** | ***p*** |
| --- | --- | --- | --- | --- |
|  | **N=91** | **N=21** | **N=70** |  |
| Age (years), x ± s | 28.7 (3.28) | 29.1 (3.41) | 28.5 (3.25) | 0.465 |
| Gestational age (weeks), median (IQR) | 19.4 (8.38) | 25.7 [19.1;28.7] | 17.7 [12.2;23.3] | 0.004* |
| Pulse (/min), x ± s | 93.7 (14.5) | 95.8 (14.0) | 93.0 (14.6) | 0.441 |
| Duration of abdominal pain (h), No. (%) |  |  |  | 0.125 |
| <24h | 48 (52.7%) | 8 (38.1%) | 40 (57.1%) |  |
| ≥24h | 43 (47.3%) | 13 (61.9%) | 30 (42.9%) |  |
| History of AA, No. (%) |  |  |  | 0.146 |
| NO | 83 (91.2%) | 17 (81.0%) | 66 (94.3%) |  |
| YES | 8 (8.79%) | 4 (19.0%) | 4 (5.7%) |  |
| Parturition, No. (%) |  |  |  | 0.278 |
| 0 | 60 (65.9%) | 14 (66.7%) | 46 (65.7%) |  |
| 1 | 30 (33%) | 6 (28.6%) | 24 (34.3%) |  |
| 2 | 1 (1.1%) | 1 (4.7%) | 0 (0.00%) |  |
| Vomiting, No. (%) |  |  |  | 0.409 |
| NO | 24 (26.4%) | 7 (33.3%) | 17 (24.3%) |  |
| YES | 67 (73.6%) | 14 (66.7%) | 53 (75.7%) |  |
| Anorexia, No. (%) |  |  |  | 0.442 |
| NO | 50 (54.9%) | 10 (47.6%) | 40 (57.1%) |  |
| YES | 41 (45.1%) | 11 (52.4%) | 30 (42.9%) |  |
| Diarrhea, No. (%) |  |  |  | 0.853 |
| NO | 77 (84.6%) | 17 (81.0%) | 60 (85.7%) |  |
| YES | 14 (15.4%) | 4 (19.0%) | 10 (14.3%) |  |
| TEMP (℃), No. (%) |  |  |  | 0.146 |
| NO | 83 (91.2%) | 17 (81.0%) | 66 (94.3%) |  |
| YES | 8 (8.79%) | 4 (19.0%) | 4 (5.7%) |  |
| Shifting pain in right lower quadrant, No. (%) |  |  |  | 0.817 |
| NO | 41 (45.1%) | 9 (42.9%) | 32 (45.7%) |  |
| YES | 50 (54.9%) | 12 (57.1%) | 38 (54.3%) |  |
| Rebound pain in right lower quadrant, No. (%) |  |  |  | 0.185 |
| NO | 28 (30.8%) | 4 (19.0%) | 24 (34.3%) |  |
| YES | 63 (69.2%) | 17 (81.0%) | 46 (65.7%) |  |
| WBC, median (IQR) | 13.8 [11.1;17.1] | 15.8 [12.6;20.5] | 12.8 [10.8;16.6] | 0.010* |
| NEUT%, median (IQR) | 87.3 [83.8;90.8] | 89.5 [86.8;94.0] | 86.0 [81.9;90.1] | 0.002* |
| CRP, median (IQR) | 24.7 [5.48;56.7] | 50.3 [22.6;68.8] | 13.6 [4.44;43.5] | 0.002* |
| NLR, median (IQR) | 10.4 [7.30;17.3] | 17.2 [10.2;24.9] | 9.85 [6.33;15.1] | 0.001* |
| TBIL, median (IQR) | 10.5 [7.80;14.2] | 13.7 [9.20;16.4] | 9.80 [7.22;13.5] | 0.011* |
| PLT, x ± s | 221 (53.1) | 214.14 (55.7) | 222.41 (52.6) | 0.535 |
| Ultrasound imaging positive, No. (%) |  |  |  | 0.436 |
| NO | 37 (40.7%) | 7 (33.3%) | 30 (42.9%) |  |
| YES | 54 (59.3%) | 14 (66.7%) | 40 (57.1%) |  |

* P＜0.05

AA, acute appendicitis; TEMP, temperature; WBC, white blood cell count; NEUT%, neutrophil percentage; CRP, C-reactive protein; NLR, neutrophil-to-lymphocyte ratio; TBIL, total bilirubin

PLT, platelet count.
