# Supplementary material for: Development of a nomogram for the prediction of complicated appendicitis during pregnancy
Source: BMC Surg. 2023 Jul 1;23:188. doi: 10.1186/s12893-023-02064-w (PMC10315032; doi:10.1186/s12893-023-02064-w)
Supplement: Supplementary file 4 — Additional File 4: Logistic regression model of the association between variables and complicated [file 12893_2023_2064_MOESM4_ESM.docx]

eTable.2 Logistic regression model of the association between variables and complicated appendicitis during pregnancy.

| **Variables** | **Univariate analysis** | |  | **Multivariate analysis** | |
| --- | --- | --- | --- | --- | --- |
|  | **OR (95% CI)** | ***P*** |  | **OR (95% CI)** | ***P*** |
| **Demographic characteristics** |  | |  |  |  |
| Age (years) | 1.06 (0.91-1.24) | 0.461 |  |  |  |
| Gestational age (weeks) | 1.08 (1.02-1.16) | **0.013** |  | 1.07(1.00-1.15) | 0.044 |
| Pulse (/min) | 1.01 (0.98-1.05) | 0.437 |  |  |  |
| History of AA |  | |  |  |  |
| NO | 1.0 (Reference) |  |  |  |  |
| YES | 3.88 (0.84-18.01) | 0.073 |  |  |  |
| **Signs and Symptoms** |  | |  |  |  |
| Duration of abdominal pain (h) |  | |  |  |  |
| ＜24h | 1.0 (Reference) |  |  |  |  |
| ≥24h | 2.17 (0.81-6.11) | 0.130 |  |  |  |
| Parturition |  | |  |  |  |
| 0 | 1.0 (Reference) |  |  |  |  |
| 1/2 | 0.96 (0.33-2.64) | 0.936 |  |  |  |
| Vomiting |  | |  |  |  |
| NO | 1.0 (Reference) |  |  |  |  |
| YES | 0.64 (0.23-1.93) | 0.411 |  |  |  |
| Anorexia |  | |  |  |  |
| NO | 1.0 (Reference) |  |  |  |  |
| YES | 1.47 (0.55-3.96) | 0.443 |  |  |  |
| Diarrhea |  | |  |  |  |
| NO | 1.0 (Reference) |  |  |  |  |
| YES | 1.41 (0.35-4.83) | 0.597 |  |  |  |
| TEMP (℃) |  | |  |  |  |
| NO | 1.0 (Reference) |  |  |  |  |
| YES | 3.88 (0.84-18.01) | 0.073 |  |  |  |

| **Variables** | **Univariate analysis** | |  | **Multivariate analysis** | |
| --- | --- | --- | --- | --- | --- |
|  | **OR (95% CI)** | **P** |  | **OR (95% CI)** | **P** |
| Shifting pain in right lower quadrant |  | |  |  |  |
| NO | 1.0 (Reference) |  |  |  |  |
| YES | 1.12 (0.42-3.07) | 0.818 |  |  |  |
| Rebound pain in right lower quadrant |  | |  |  |  |
| NO | 1.0 (Reference) |  |  |  |  |
| YES | 2.22 (0.72-8.36) | 0.192 |  |  |  |
| **Laboratory examinations** |  | |  |  |  |
| WBC | 1.1 (1-1.22) | 0.074 |  |  |  |
| NEUT% | 1.19 (1.07-1.35) | **0.004** |  | 1.17(1.05-1.34) | 0.012 |
| CRP | 1.02 (1-1.03) | **0.012** |  | 1.02(1.00-1.03) | 0.018 |
| NLR | 1.04 (1.01-1.1) | 0.079 |  |  |  |
| TBIL | 1.12 (1.03-1.24) | **0.013** |  |  |  |
| PLT | 1 (0.99-1.01) | 0.530 |  |  |  |
| **Ultrasound imaging positive** |  | |  |  |  |
| NO | 1.0 (Reference) |  |  |  |  |
| YES | 1.5 (0.55-4.38) | 0.437 |  |  |  |

eTable.2 Logistic regression model of the association between variables and complicated appendicitis during pregnancy. (continued)

AA, acute appendicitis; TEMP, temperature; WBC, white blood cell count; NEUT%, neutrophil percentage; CRP, C-reactive protein; NLR, neutrophil-to-lymphocyte ratio; TBIL, total bilirubin

PLT, platelet count.
